# Role of Imaging Studies in Evaluating Patients Post Cytoreductive Surgery and Hyperthermic Intraperitoneal Chemotherapy

**DOI:** 10.7759/cureus.20601

**Published:** 2021-12-22

**Authors:** Mohamed Fayed, Santhalakshmi Angappan, Oghenekpaobor Oyibo, Arif Valliani

**Affiliations:** 1 Anesthesiology and Perioperative Medicine, Henry Ford Health System, Detroit, USA; 2 Anesthesiology, Perioperative Medicine and Pain Management, Henry Ford Health System, Detroit, USA; 3 Anesthesiology, Henry Ford Health System, Detroit, USA

**Keywords:** abdominal imaging, abdominal radiology, physical exam, abdomino-pelvic ct, abdominal x-ray, free air in the abdomen, silent perforation of bowel, bowel obstruction, mesenteric ischemia, cytoreductive surgery and hipec

## Abstract

A 77-year-old male presented to the ED with a new onset of acute abdominal pain, nausea, and vomiting. He had a previous surgical history of cytoreductive surgery (CRS) and hyperthermic intraperitoneal chemotherapy (HIPEC) for an appendiceal tumor. Despite the repeated reassuring abdominal examinations, CT abdomen showed high-grade bowel obstruction and perforation. He was urgently taken to the operating room and underwent resection of 70 cm segment of small ischemic bowel with primary anastomosis. His postoperative course was complicated with atrial fibrillation (AF) requiring cardioversion and medical therapy. Later, he was discharged home under stable conditions. Relying on abdominal signs, an abdominal exam in a patient with a previous history of extensive peritonectomy and post-HIPEC surgery is challenging due to the altered peritoneal anatomy. As a result, the abdominal examination findings can be benign and misleading. This can lead to delayed surgical intervention, thereby increasing morbidity and mortality significantly. Therefore, a detailed evaluation with a low threshold for abdominal imaging studies like abdominal X-rays and CT abdomen series is warranted in this subset of patients.

## Introduction

Primary peritoneal cancers are rare and mainly occur secondary to disseminating tumor cells from the primary GI or gynecological cancer [1]. Most often, they are associated with ascites and lymphadenopathy. The mainstay treatment for this secondary extensive peritoneal cancer is cytoreductive surgery (CRS) combined with hyperthermic intraperitoneal chemotherapy (HIPEC). This treatment has been shown to improve survival rates [2,3]. However, CRS and HIPEC treatment are complex surgical procedures involving total peritonectomy if metastasis is extensive [4]. In addition, repeated lavages in the peritoneal cavity with high-dose thermo-chemotherapeutic agents could amplify the peritoneal irritation and inflammation. This, in turn, can contribute to the development of complex adhesions, distorted anatomy, destruction of the visceral peritoneum and its nerve supply, and severe postoperative pain after surgery [5].

Although HIPEC, as a treatment for extensive metastasis, was established almost a century ago, the procedure itself has evolved over several years and has been opted more frequently over the last few years. Hence, the available literature on HIPEC and its long-term complications is not well elucidated. In addition, the long-term outcomes of patients older than 70 years old post CRS and HIPEC procedures are not available in the literature.

We report a case of mucinous appendiceal cancer treated with HIPEC and CRS three years prior. He presented with bowel ischemia despite repeated reassuring abdominal exams, thereby making the reliability of abdominal physical exams questionable in these situations.

## Case presentation

A 77-year-old man presented to the emergency department with acute abdominal pain of one-day duration. He had a past medical history of chronic atrial fibrillation (AF), obstructive sleep apnea, and mucinous appendiceal cancer status post CRS including right hemicolectomy, pelvic peritonectomy, omentectomy, and HIPEC three years prior.

**Figure 1:**
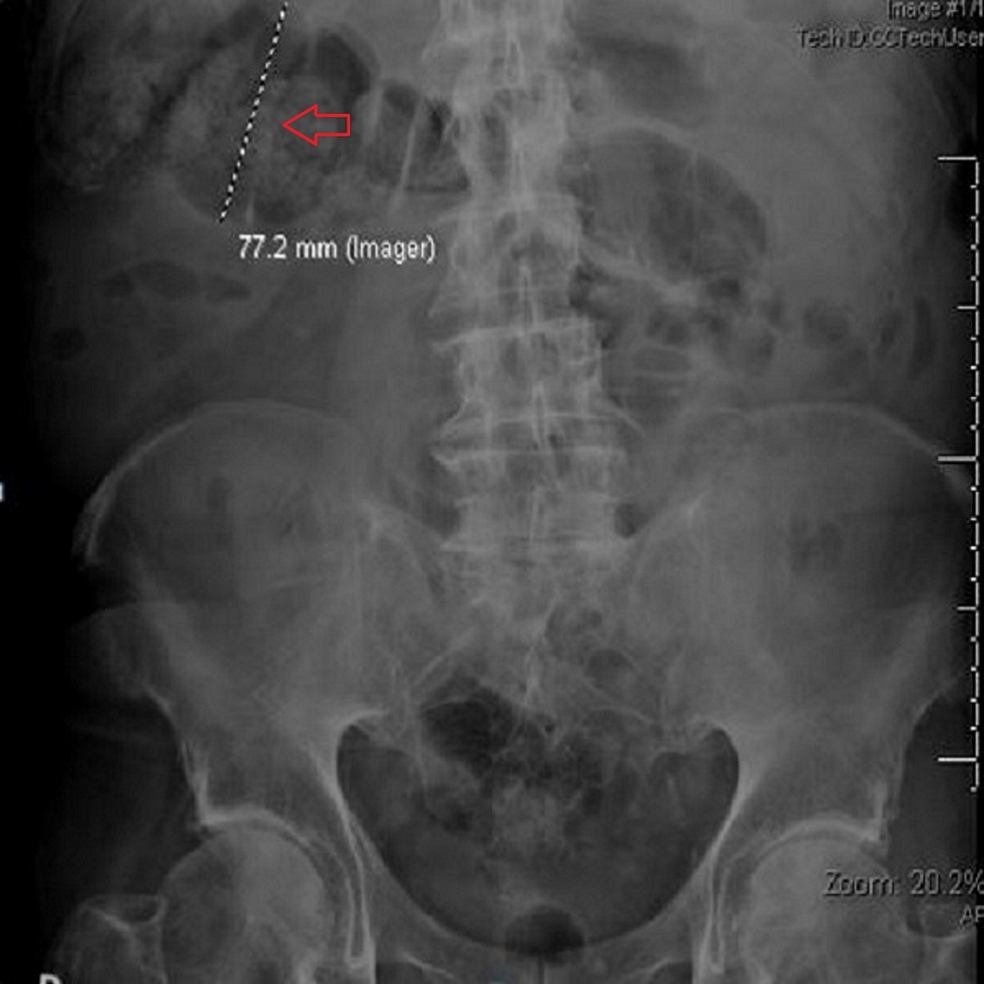
Abdominal X-ray of our patient. Red arrow: Dilated transverse colon, 7.7 cm in diameter.

The pain was located around the epigastrium. It was non-radiating, 7 out of 10 in intensity, with persistent belching, and associated with nausea and vomiting. On admission, he was afebrile, normotensive, with an oxygen saturation of 96% on room air. Abdominal examination showed a soft and non-tender abdomen. Laboratory work was significant for mild hyponatremia, white cell count of 11.8 k/mm3, neutrophil count of 86%, hemoglobin 13.5 gm/dL, and platelets 193 k/mm3 with normal liver function tests, lipase, and urine analysis. Given his persistent nausea and vomiting, the abdominal X-ray showed mild colonic dilatation with scattered air-fluid levels consistent with an ileus (Figure 1). The patient was kept fasted; he received nasogastric decompression of his stomach and IV fluids.

**Figure 2:**
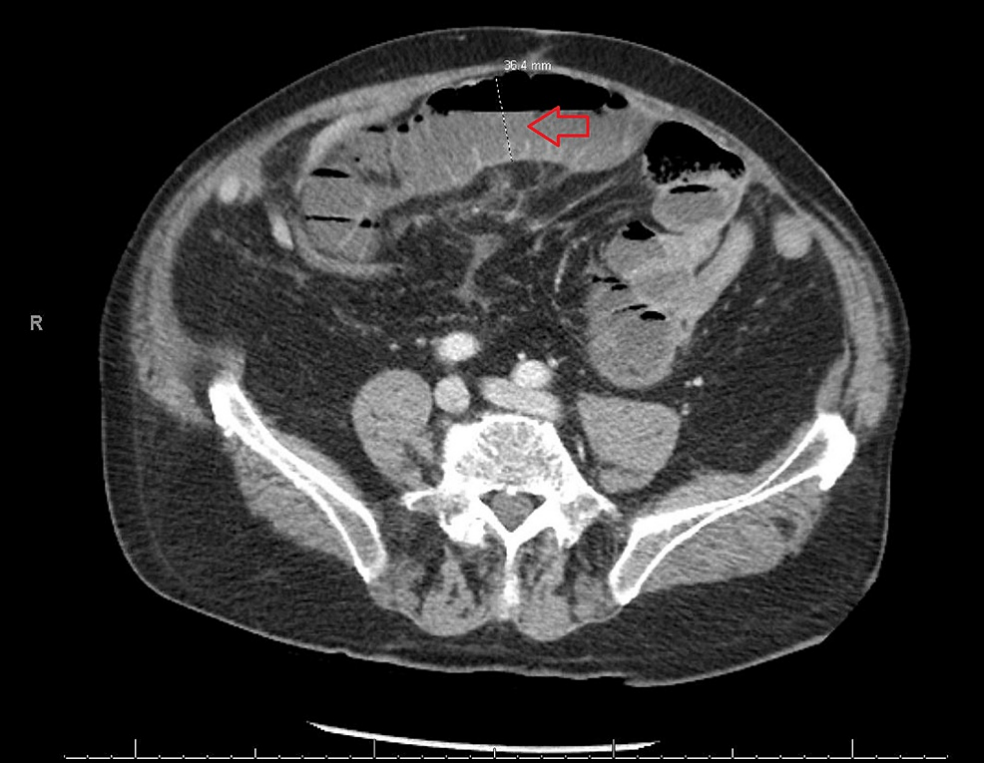
CT scan of the abdomen. Red arrow: Dilated small bowel loop, 4 cm in diameter.

**Figure 3:**
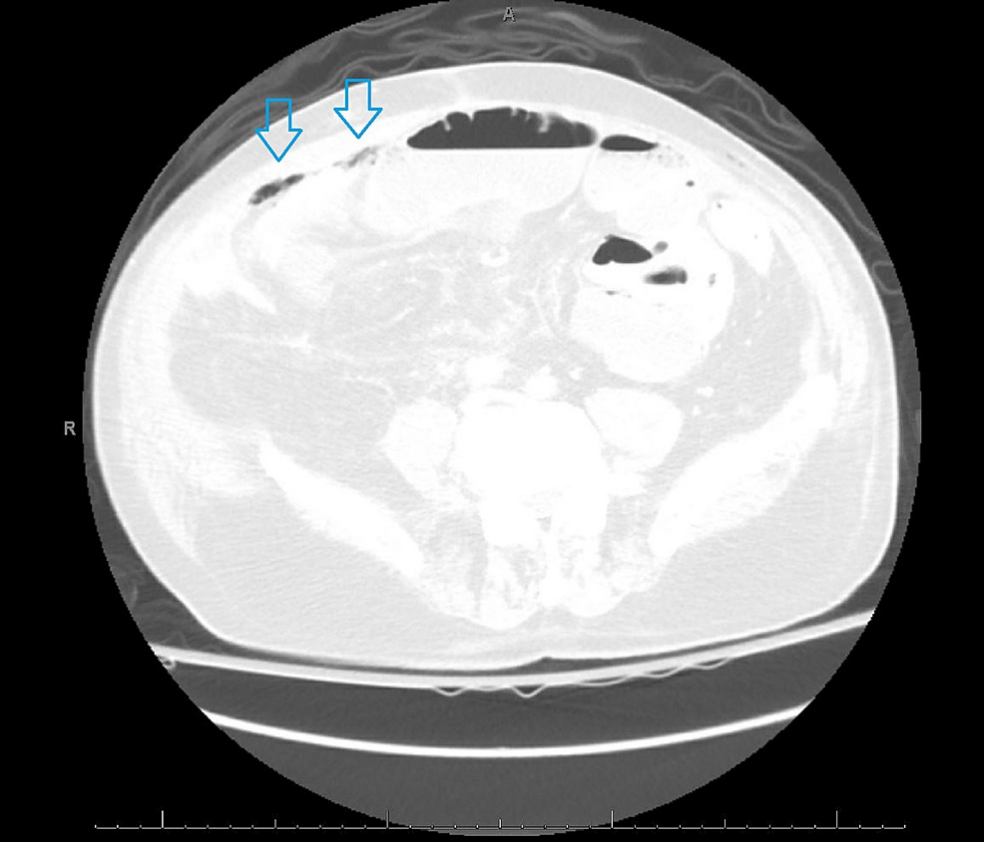
CT scan of the abdomen, lung window level. Blue arrow: Pockets of free air seen on lung window.

However, over the next 24 hours, the patient still had an unremarkable abdominal exam with no improvement of his symptoms, and his AF rate was not controlled. CT scan of his abdomen and pelvis showed high-grade partial small bowel obstruction with a transition point in the proximal to mid ileum and free air in the peritoneum (Figures 2-3).

The patient underwent exploratory laparotomy with resection of 70 cm segment of the small necrotic bowel. In addition, he received IV cefepime for *Escherichia coli* bacteremia. Later, due to ongoing hemodynamic instability, AF with a rapid ventricular response, and distended abdomen, re-exploration was done three days later with the removal of another 20 cm of small intestine with a total remaining small bowel length of 140 cm. Following his surgeries, he was started on total parenteral nutrition with gradual up-titration of caloric requirements.

The patient's hospital course was complicated by intraabdominal fluid collection requiring interventional radiology guided drain placement and uncontrolled AF with rapid ventricular response requiring amiodarone and electrical cardioversion. After a month of prolonged hospital stay, the patient was discharged on a liquid diet and supplemental parenteral nutrition secondary to short bowel syndrome, with outpatient follow-up with a nutritionist and primary care provider.

## Discussion

CRS and HIPEC surgeries can significantly influence the overall disease-free survival in selected patients suffering from peritoneal surface malignancies (PSM) [6]. The most common causes for post HIPEC complications and readmissions include failure to thrive, infection, and ileus and bowel obstruction (Table 1) [7,8]. Our patient presented three years after his initial surgery decreasing the likelihood of these complications being the cause of his symptoms. For transmural bowel necrosis, the risk factors in our patient are embolic acute mesenteric ischemia from chronic AF and small-bowel obstruction from previous CRS and HIPEC [9].

**Table 1:** Incidence of complications after hyperthermic intraperitoneal chemotherapy.

Cardiac complications	Incidence of 8%
Pulmonary embolism	0.9%
Pneumonia	5.4%
Pneumothorax	5.4%
Ileus	60%
Fistula	1.8%
GI abscess	7.3%
Anastomotic leak	9.1%
Hemorrhage	8.1%
Acute kidney injury	5.4%
Sepsis	6.4%
Superficial skin infection	5.4%

Our differential diagnoses included gastroenteritis, peptic ulcer disease, pancreatitis, renal colic, mesenteric ischemia, and small bowel obstruction. Although patient laboratory results were significant for leukocytosis, the patient denied a history of diarrhea, fever, or bloody stool. Also, given the fact that his symptoms were refractory to medical management, we ruled out gastroenteritis and peptic ulcer disease. His amylase and lipase were within normal limits. Nephrolithiasis was also ruled out as urine analysis did not show evidence of RBCs, and there was no costophrenic angle tenderness. Due to persistent symptoms, we ended up doing a CT scan of the abdomen, which showed mesenteric ischemia. 

There are few explanations to consider for the repeated unremarkable abdominal exam. First, our patient had total peritonectomy during his CRS. Abdominal tenderness and rebound tenderness rely on the innervation of parietal and visceral peritoneum. Parietal peritoneum is innervated by afferent fibers from lower intercostal nerves and lumbar plexus. It is sensitive to sharp pain, pressure, and temperature. However, visceral peritoneum only receives its innervations via the autonomic nervous system and perceives dull pain sensation [4]. With extensive and total peritonectomy, there is no peritoneum left to elicit inflammation of the peritoneum, thereby demonstrating negative abdominal signs on exam. Secondly, chemotherapy-induced polyneuropathy is one of the most common side effects of cancer treatment [10]. It can lead to permanent symptoms and disability in up to 40% of cancer survivors [11]. Finally, our patient had HIPEC with mitomycin, which can alter nerve conduction and pain perception at high concentrations. It has been shown that high concentrations of mitomycin injection can reduce the thickness of the sciatic nerve myelin sheath compared with lower concentrations [12]. Given the altered peritoneal anatomy and innervation, the inability to localize his abdominal pain made it challenging to narrow down the diagnosis.

Clinical investigations are crucial to support the diagnosis and guide appropriate medical management. In the setting of acute abdomen, leukocytosis, electrolyte derangements, and lactic acidosis may be suggestive of many causes, including sepsis, bowel ischemia, or perforation [13]. Furthermore, imaging studies like abdominal X-ray series are quick, low-cost, and 50%-70% sensitive in identifying even small amounts of pneumoperitoneum and small and large bowel obstructions [9]. However, CT scans of the abdomen and pelvis are the most sensitive and specific tests to diagnose a perforation and ascertain the most likely etiology. CT abdomen is reported to have a sensitivity of 78%-100% for the detection of complete or high-grade small bowel obstruction. However, it may not allow accurate diagnosis in cases involving incomplete obstruction. CT scan also allows the visualization of the transition point, the severity of obstruction, potential etiology, and assessment of any life-threatening complications [13]. CT scan of our patient showed evidence of bowel ischemia and perforation.

## Conclusions

Patients who underwent extensive or total peritonectomy as a part of the CRS and intraoperative HIPEC can present with atypical signs and symptoms of acute abdomen. This can lead to a significant delay in diagnosis and management, adversely affecting the outcomes. Hence, there should be a low threshold for imaging modalities like x-ray abdomen, CT abdomen, and CT angiogram in case of atypical presentations with high suspicion for small bowel obstruction or acute mesenteric ischemia. This is done to avoid any delay in the surgical intervention, which can negatively influence patients to increase the length of stay in the hospital, morbidity, and mortality significantly.
